# Baicalein Induces Apoptosis of Pancreatic Cancer Cells by Regulating the Expression of miR-139-3p and miR-196b-5p

**DOI:** 10.3389/fonc.2021.653061

**Published:** 2021-04-30

**Authors:** Danhui Ma, Sinuo Chen, Heming Wang, Jiayi Wei, Hao Wu, Hong Gao, Xinlai Cheng, Taotao Liu, Shi-Hua Luo, Yicheng Zhao, Guangqi Song

**Affiliations:** ^1^ Department of Gastroenterology and Hepatology, Zhongshan Hospital of Fudan University, Shanghai, China; ^2^ Shanghai Institute of Liver Diseases, Shanghai, China; ^3^ Buchmann Institute for Molecular Life Sciences, Goethe University Frankfurt, Frankfurt, Germany; ^4^ Department of Traumatology, Rui Jin Hospital, School of Medicine, Shanghai Jiao Tong University, Shanghai, China; ^5^ Clinical Medical College, Changchun University of Chinese Medicine, Changchun, China

**Keywords:** baicalein, pancreatic cancer, microRNA, high-throughput sequencing, apoptosis

## Abstract

Pancreatic cancer is a common malignant tumor with a high incidence and mortality rate. The prognosis of patients with pancreatic cancer is considerably poor due to the lack of effective treatment in clinically. Despite numerous studies have revealed that baicalein, a natural product, is responsible for suppressing multiple cancer cells proliferation, motility and invasion. The mechanism by which baicalein restraining pancreatic cancer progression remains unclear. In this study, we firstly verified that baicalein plays a critical role in inhibiting pancreatic tumorigenesis *in vitro* and *in vivo*. Then we analyzed the alteration of microRNAs (miRNAs) expression levels in Panc-1 cells incubated with DMSO, 50 and 100 μM baicalein by High-Throughput sequencing. Intriguingly, we observed that 20 and 39 miRNAs were accordingly up- and down-regulated through comparing Panc-1 cells exposed to 100 μM baicalein with the control group. Quantitative PCR analysis confirmed that miR-139-3p was the most up-regulated miRNA after baicalein treatment, while miR-196b-5p was the most down-regulated miRNA. Further studies showed that miR-139-3p induced, miR-196b-5p inhibited the apoptosis of Panc-1 cells *via* targeting NOB1 and ING5 respectively. In conclusion, we demonstrated that baicalein is a potent inhibitor against pancreatic cancer by modulating the expression of miR-139-3p or miR-196b-5p.

## Introduction

Pancreatic cancer is one of the most malignant cancers with relatively high incidence and mortality (1). According to the statistical data of GLOBOCAN 2012, there were about 300,000 pancreatic cancer patients in this single year, and almost the same number of deaths caused by this disease worldwide because of its high degree of malignant transformation and the difficulties in early diagnosis (2). Pancreatic cancer mainly includes two types, exocrine cancer (such as adenocarcinoma) and neuroendocrine cancer. The former type accounts for the majority of cases and has always been emphasized in clinical and basic investigation (3). Although surgery, chemotherapy and radiotherapy have already been adopted for the treatment of pancreatic cancer, the prognosis of which was still extremely poor (4–6). Furthermore, the etiology and pathogenesis of pancreatic cancer have not been fully understood. In order to better treat pancreatic cancer, high curative-effect and low side-effect targeting drugs are urgent to be exploited as soon as possible.

Baicalein, a main active flavonoid ingredient purified from *Scutellaria baicalensis Georgi*, has been found to have significant therapeutic potentials in inflammatory diseases, neurological disorders and tumors (7–12). Several studies demonstrated that baicalein has the ability to inhibit the proliferation, invasion, migration and adhesion of various types of cancers (13–16). Yan et al. found that baicalein promoted cell apoptosis and autophagy through down-regulating PI3K/AKT pathway in breast cancer (17). Baicalein was also proved to cause PD-L1 suppression mediated by restraining STAT3 activity in hepatocellular carcinoma (18). Besides, another study in 2018 showed that baicalein was able to regulate the epithelial–mesenchymal transition (EMT) by targeting tumor associated macrophages (19). Although existing studies partially revealed the potential mechanism of baicalein inhibiting the progression of different cancers, the detailed function of baicalein performing in pancreatic cancer still remains unclear.

miRNAs are small endogenous non-coding RNAs with approximately 22 nts, which could regulate the translation and cleavage of mRNA (20–22). A single miRNA is able to bind to a variety of specific mRNAs and gives play to gene silencing and transcriptional inhibition (23). MiRNAs are responsible for many biological processes, such as cell proliferation, apoptosis, fat metabolism, migration and invasion (24–27). Previous studies found that the modulation of miRNAs expression level by baicalein was closely related to its obviously biological effects (28, 29). Baicalein decreased the expression level of miR-424-3p in lung cancer to suppress cell proliferation and improve cisplatin sensitivity (13). Baicalein activates p38-MAPK-JNK pathway *via* increasing the expression level of miR-29 to retard proliferation and collagen deposition (30). To date, few studies reported whether baicalein could alter several specific miRNAs expression pattern to further affect the progression of pancreatic cancer.

In our study, we firstly treated Panc-1 cells with equivalent DMSO, 50 and 100 μM baicalein. The results suggested that baicalein not only prominently inhibited cell proliferation, motility and invasion, but induced cell cycle arrest in S phase and promoted apoptosis. Through analyzing the High-Throughput sequencing results of miRNAs profiling between the DMSO group and 100 μM baicalein treated group, we discovered that the expression level of 20 miRNAs were up-regulated and 39 miRNAs were down-regulated. Intriguingly, the value of fold change was related to the concentration of baicalein. Furthermore, we verified that miR-139-3p, the most significant up-regulated miRNA, promotes apoptosis of Panc-1 cells *via* targeting NOB1. miR-196b-5p, the most significant down-regulated miRNA, restrains apoptosis of Panc-1 cells *via* targeting ING5. In brief, we demonstrated that the alteration of miRNAs profiling induced by baicalein is crucial for suppressing the progression of pancreatic cancer.

## Materials and Methods

### Cell and Reagents

Panc-1 cells were preserved in our laboratory and were cultured in high-glucose Dulbecco’s modified Eagle’s medium (DMEM, Hyclone) containing 10% fetal bovine serum (FBS, Gibco) and 1% penicillin–streptomycin (Hyclone). All cells were cultured in a humidified incubator with 5% CO_2_ at 37 °C. Trypsin (Hyclone) was used to dissociate cells. Baicalein (purity > 98%) was purchased from Institute for the Control of Pharmaceutical and Biological Products (Beijing, China). Dimethyl sulphoxide (DMSO) was purchased from SigmaAldrich (St. Louis, MO, USA). Antibodies against β-actin (ab8226), cleaved caspase-3 (ab49822), p21 (ab109520), CCND1 (ab16663), NOB1 (ab205718) and ING5 (ab259904) were purchased from Abcam.

### RNA Extraction and Quantitative RT-PCR (qRT-PCR)

After Panc-1 cells grew to a certain confluency, they are treated with three concentrations of baicalein (0, 50, 100 μM) for 72 h. Cells were then collected after digestion and washed once with PBS. Total RNA was extracted using Trizol reagent (Invitrogen) according to the standard RNA isolation protocol. The concentration of RNA was measured by NanoDrop 8000 spectrophotometer (Thermo Fisher Scientific). Single-stranded complementary DNA was synthesized from per 500 ng RNA in a 10 μL reaction volume with reverse transcription kits (Takara), and the reaction was performed according to the manufacturer’s protocol. qRT-PCR was carried out using a SYBR Green PCR kit (Thermo Fisher Scientific) following the protocol provided by the manufacturer and the cycle threshold (Ct) of each gene was recorded. The U6 small nuclear RNA was used as internal reference to calculate miRNAs expression and GAPDH was used as internal reference to calculate Caspase-3, p21, CCND1, NOB1 and ING5 expression. Data were analyzed by the comparative Ct method (2^−ΔΔCt^) (31). The primers used in this study were shown in **Table S2** (shown in **Supplementary Material**).

### Construction of MiRNAs Libraries and MiRNAs Expression Analysis

Total RNA was extracted by mirVana™ miRNAs Isolation Kit. After treatment with DNase I, 1 µg extracted RNA of each sample was taken to construct the small RNA library according to NEBNext^®^ Multiplex Small RNA Library Prep Set for Illumina^®^. The concentration of each sample was determined using NanoDrop 8000 spectrophotometer (Thermo Fisher). Then libraries of small RNAs were sequenced by Illumina Novaseq 6000 (2 × 150 bp paired-end). The sequencing raw data was submitted to Sequence Read Archive (SRA), and the accession number is PRJNA690773. To get clean data, we used Trim_galore (0.6.4) to remove the adapter sequences from the raw data and filtered out sequences with QC < 30. The clean data was mapped by Bowite (1.0.0) to miRBase (Release 22) (www.mirbase.org), and then used Samtools (1.7) to calculate the counts of miRNAs. Different miRNA analysis was analyzed by DESeq2. The target gene of miRNAs was derived from miRTarBase (http://mirtarbase.mbc.nctu.edu.tw/index.html) (32) and the network diagram of miRNA and target gene was drawn by Cytoscape. R-Package clusterProfiler (33) was used to analyze the target genes of miRNAs for KEGG and GO enrichment analysis.

### MiRNA Transfection

When cell confluency met approximately 60–80%, cells in six-well plate were transfected with miRNA mimics or inhibitors as well as negative control miRNA using Lipo-fectamine^TM^ 2000 (Invitrogen). MiRNA mimics or inhibitors purchased from Shanghai GenePharma Co., Ltd. After transfection 6–8 h, medium was changed by fresh DMEM containing 10% FBS and cells were harvested after 24–72 h.

### Cell Proliferation and Morphological Examination

After digestion, the cells were seeded in a six-well plate with a quantity of 2 × 10^5^ per well. After overnight incubation, the medium containing with equivalent DMSO, 50 μM baicalein and 100 μM baicalein was added, and the cell morphology was photographed at 0 h. After that, the cell morphology in different groups were photographed at 48 h. At 72 h, all groups of cells were digested and then counted from three individual experiments to value the activity of cell proliferation.

### CCK-8 Assay

The viability of cells treated with DMSO and different concentration of baicalein (50 and 100 μM) were obtained with Cell Counting Kit 8 (Beyotime Biotechnology). 2 × 10^3^ cells per well were seeded in 96-well plates treated with different concentration of baicalein. All these cells were cultured for the indicated times (0, 24, 48 and 72h), and the cell viability was measured per 24 h stimulation by a multifunctional reader (MD FlexStation 3) to detect the absorbance of the cells at 450 nm according to the manufacturer’s instructions.

### Transwell Assay

2 × 10^4^ cells were harvested in serum-free medium and then seeded in 200 μL serum-free DMEM onto transwell chambers (Corning) with the lower part filled with 600 μl DMEM containing 20% FBS. Meanwhile, DMSO and 100 μM baicalein were added to cells, which were cultured for 48 h at 37 °C and were fixed in 4% para-formaldehyde and stained with 0.1% crystal violet. Each group had three independent duplications.

### Wound Healing Assay

8 × 10^5^ cells per well were dissociated and seeded in 6-well plates. After overnight incubation, the cell monolayer in each well was scratched using a plastic tip vertically across the plate and then washed twice with PBS until no suspending cells were observed in the wound areas under the microscope. Subsequently, the cells were divided into three groups, each containing three replicates and were incubated in serum-free medium with different concentration of baicalein (0, 50, 100 μM) and images were taken at 0 and 48 h to measure the distance of wound.

### Clone Formation Assay

2 × 10^3^ cells per well were seeded onto 6-well plates and were cultured for 7–10 days until visible clone were formed and stained with crystal violet solution (0.1% crystal violet, 25% methanol in ddH_2_O). The clones were recorded by camera and were counted by ImageJ. Each group had three independent repeats.

### Apoptosis Assay

Apoptotic cells were determined with an Annexin V–fluorescein isothiocyanate (FITC)/PI apoptosis detection kit (Beyotime Biotechnology) according to the manufacturer’s instructions. Cells were incubated with baicalein for 72 h and were measured by flow cytometry and each group had three repeats.

### Cell Cycle

After laying 6-well plates with 8 × 10^5^ cells per well, cells were cultured in medium containing three concentrations of baicalein (0, 50, 100 μM) for 72 h. Cell cycle were detected by a cell cycle and apoptosis kit (Beyotime Biotechnology) and were measured by flow cytometry according to standard instructions. The rates of cell cycle were computed by ModFit LT software.

### Xenograft Tumor Model

All animal investigation in our study was conformed to the guidelines of Animal Care and Use Committee, Zhongshan Hospital of Fudan University. Balb/c nude mice were purchased from Vital River Laboratory Animal Technology Co., Ltd. 1 × 10^6^ Panc-1 cells were subcutaneously injected into 4 weeks old female mice. After a week, mice bearing tumors were randomly divided into two groups and each group consisted of five mice. Then mice were administered *via* intraperitoneal injection control solvent (5% DMSO and 95% saline) or 10 mg/kg baicalein (dissolved in 5% DMSO and 95% saline) thrice a week for 4 weeks (34). Body weights of mice were measured every week. Tumor volumes were measured by the formula *V* = (*a* × *b* ^2^)/2 (*V* is the tumor volume, *a* is the length of the tumor, *b* is the width of the tumor).

### Statistical Analysis

All data are showed as the mean ± SD (standard deviation). GraphPad 7.0 was used for data analysis. The unpaired, two-tailed Student’s t test was used to compare the significance of differences between experimental groups and controls from at least three independent repeats. ****p <0.0001, ***p <0.001, **p <0.01, *p <0.05, N.S. means no significance.

## Results

### Baicalein Inhibits Proliferation, Motility and Invasion of Pancreatic Cancer Cells

**Figure 1:**
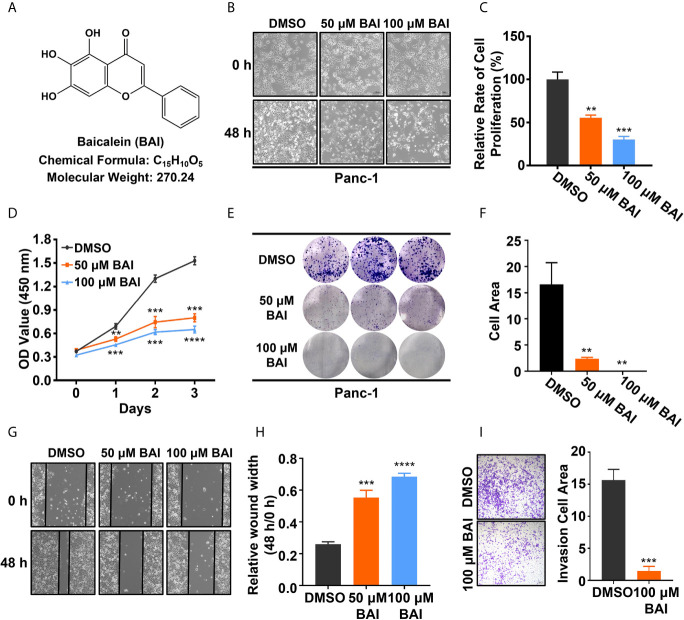
Baicalein inhibits proliferation, motility and invasion of pancreatic cancer cells *in vitro.* **(A)** Structural formula of baicalein (BAI). **(B)** The morphology of Panc-1 cells treated with DMSO, 50 µM and 100 µM baicalein for 0 and 48 h. **(C)** The relative cell proliferation rate of Panc-1 cells treated with DMSO, 50 µM and 100 µM baicalein for 72 h. **(D)** The cell viability of Panc-1 cells treated with DMSO, 50 µM and 100 µM baicalein for 0, 24, 48 and 72 h. **(E, F)** The clone formation of Panc-1 cells treated with DMSO, 50 µM and 100 µM baicalein. Cell area was measured by ImageJ. **(G, H)** The wound healing assay of Panc-1 cells treated with DMSO, 50 µM and 100 µM baicalein for 48 h. The value of relative wound width was measured by ImageJ. **(I)** Transwell assay of Panc-1 cells treated with DMSO and 100 µM baicalein for 48 h. Cell area was measured by ImageJ. ** means P < 0.01, *** means P < 0.001, **** means P < 0.0001.

To verify whether baicalein (chemical formula as shown in **Figure 1A**, shortly BAI) functions in pancreatic cancer *in vitro*, equivalent DMSO, 50 μM baicalein and 100 μM baicalein was respectively added into Panc-1 cells for 48 h. The microscope records suggested that the density of Panc-1 cells in baicalein-treated group at 48 h was markedly less than the control group (**Figure 1B**). After treatment with baicalein for three days, the statistical result showed that baicalein significantly inhibited the proliferation of Panc-1 cells in a concentration dependent manner (**Figure 1C**). In addition, CCK-8 assay was performed to measure the cell viability of Panc-1 cells exposing to DMSO, 50 μM baicalein or 100 μM baicalein. The result also suggested that baicalein could restrain the viability of Panc-1 cells (**Figure 1D**). Then we performed colony formation experiment and measured the results by ImageJ, which indicated that baicalein decreased the ability of clone formation of Panc-1 cells (**Figures 1E, F**). Besides, wound healing assay demonstrated that baicalein indeed inhibited the ability of cell motility (**Figures 1G, H**). Transwell results showed that there was a significant difference in the number of migrated cells between the DMSO group and 100 μM baicalein group, which suggested baicalein was able to inhibit the invasion of Panc-1 cells (**Figure 1I**). Taken together, these results showed that baicalein isolated from natural product obviously inhibits proliferation, motility and invasion of pancreatic cancer cells *in vitro*.

### Baicalein Inhibits Tumorigenesis of Pancreatic Cancer *In Vivo*

**Figure 2:**
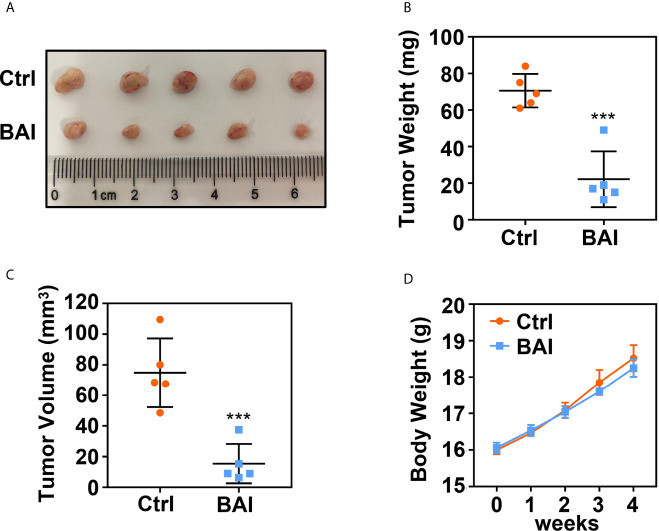
Baicalein inhibits tumorigenesis of pancreatic cancer. **(A)** Xenograft tumor formation of the control (Ctrl) group (5% DMSO and 95% saline) and Baicalein (BAI) group (10 mg/kg BAI, dissolved in 5% DMSO and 95% saline). Each group consisted of five mice. **(B)** Tumor weights of the Ctrl group and BAI group. **(C)** Tumor volumes of the Ctrl group and BAI group. **(D)** Body weights of the Ctrl group and BAI group. *** means P < 0.001.

To address whether baicalein could inhibit pancreatic cancer cell proliferation *in vivo*, we took advantage of xenograft tumor model in our study. 1 × 10^6^ Panc-1 cells were subcutaneously injected into 4 weeks old female Balb/c nude mice. Mice bearing tumor were randomly divided into two groups, the control group (5% DMSO and 95% saline) and the baicalein group (10 mg/kg, dissolved in 5% DMSO and 95% saline). Baicalein and corresponding solvent were administered *via* intraperitoneal injection thrice a week for 4 weeks. Tumor weights and volumes were recorded at the end of treatment. The results suggested that baicalein obviously decreased pancreatic tumor weight and volume *in vivo* (**Figures 2A–C**). In addition, we found that there was no significant difference in mice body weights between the control group and baicalein treated group, which indicated that baicalein nearly has no toxicity to mice (**Figure 2D**).

### Baicalein Induces Apoptosis and Cell Cycle Arrest in Panc-1 Cells

**Figure 3:**
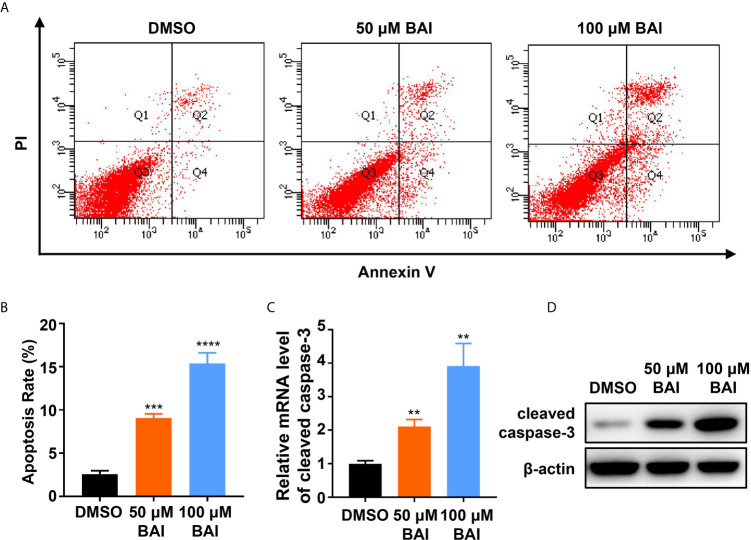
Baicalein induces apoptosis in a concentration dependent manner. **(A)** Apoptosis results of Panc-1 cells treated with DMSO, 50 µM and 100 µM baicalein for 48 h by flow cytometry. Q1 represents death cells, Q2 represents the late apoptosis cells, Q3 represents the normal cells, Q4 represents the ealry apoptosis cells. **(B)** The statistical result of apoptosis rate, which equals to the rate of late apoptosis cells (Q2) plus the rate of early apoptosis cells (Q4). **(C, D)** The mRNA **(C)** and protein **(D)** level of apoptosis related protein cleaved caspase-3 in Panc-1 cells treated with DMSO, 50 µM and 100 µM baicalein. ** means P < 0.01, *** means P < 0.001, **** means P < 0.0001.

To determine the mechanism of how baicalein inhibits pancreatic cells proliferation, we treated Panc-1 cells with DMSO, 50 μM baicalein or 100 μM baicalein for 48 h and performed Annexin V-FITC/PI assay to detect apoptosis rate by flow cytometry. The statistic result showed that the proportion of cells in early and late apoptosis was significantly increased after the treatment of baicalein and higher concentration of baicalein contributes to higher apoptosis rate (**Figures 3A, B**). We also examined the mRNA and protein level of Cleaved caspase-3 in Panc-1 cells treated with DMSO, 50 µM and 100 µM baicalein by qPCR and western blotting assay. Cleaved caspase-3 as the activated form of caspase-3 plays a key role in the pathway of apoptosis. The results suggested that baicalein significantly increases the expression of cleaved caspase-3 in a dosage dependent manner (**Figures 3C, D**).

**Figure 4:**
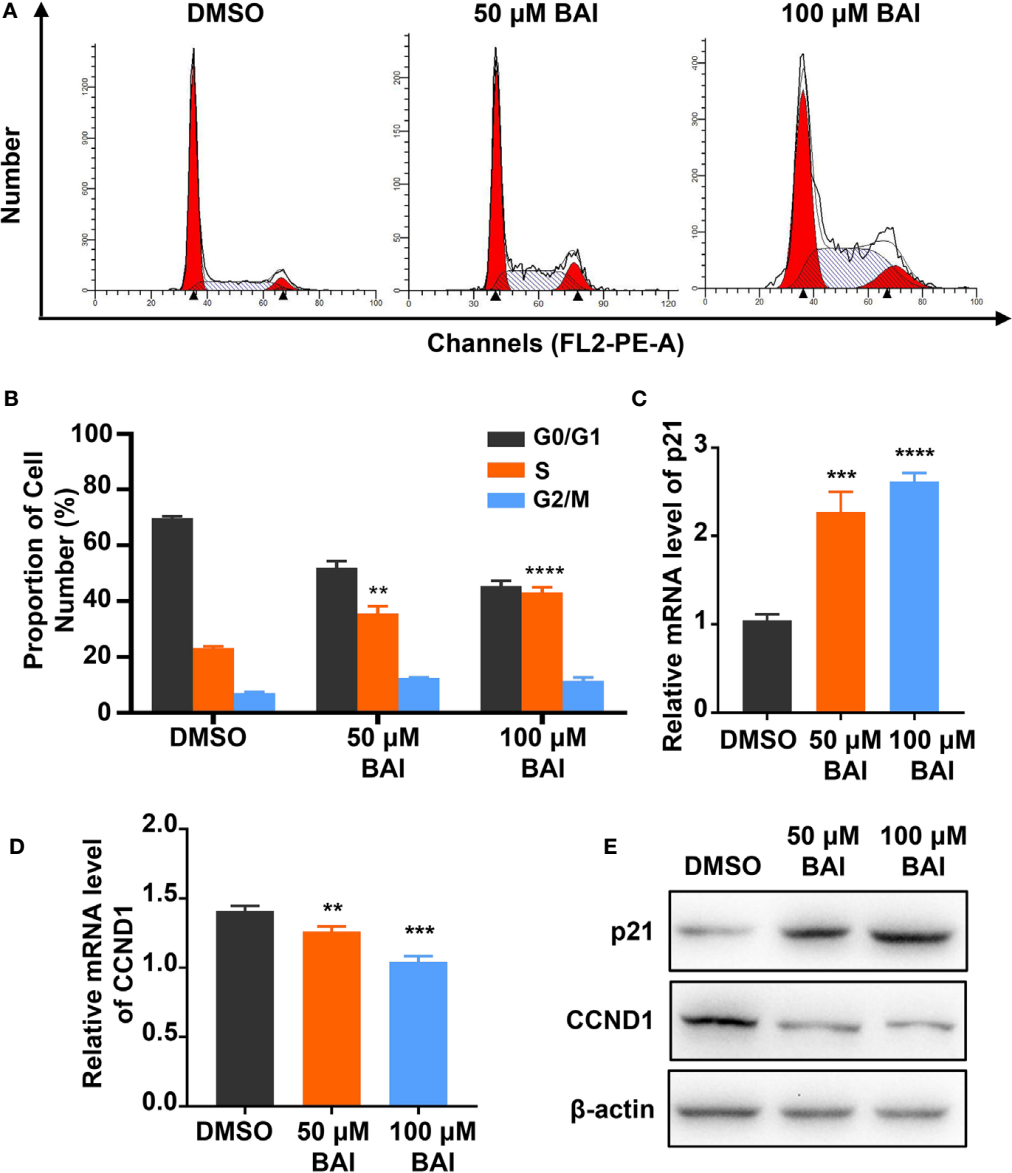
Baicalein induces cell cycle arrest in S phase. **(A)** Cell cycle results of Panc-1 cells treated with DMSO, 50 µM and 100 µM BAI for 48 h by flow cytometry. **(B)** The statistical results of cell number measured by ModFit LT software. **(C, D)** The relative mRNA level of cell cycle related genes p21 **(C)** and CCND1 **(D)** measured by qPCR. **(E)** The protein level of p21 and CCND1 in Panc-1 cells treated with DMSO, 50 µM and 100 µM BAI measured by western blotting. ** means P < 0.01, *** means P < 0.001, **** means P < 0.0001.

To further confirm whether baicalein is able to affect cell cycle, we detected the proportion of cell phases through flow cytometry in Panc-1 cells treated with DMSO, 50 μM or 100 μM baicalein for 72 h. The results showed that baicalein induced S phase arrest in a concentration dependent manner (**Figures 4A, B**). Then we examined the mRNA and protein level of cell cycle related genes p21 and cyclinD 1 (CCND1) by qPCR and western blotting. We found that baicalein obviously increased p21 and decreased CCND1 levels in a dose-dependent manner (**Figures 4C–E**), which suggests that baicalein inhibit cell proliferation by extending duration of S phase. As a result, we concluded that baicalein inhibited pancreatic cells proliferation through promoting apoptosis and cell cycle arrest in S phase.

### Alteration of MiRNAs Profiling in Panc-1 Cells Treated With Baicalein

**Figure 5:**
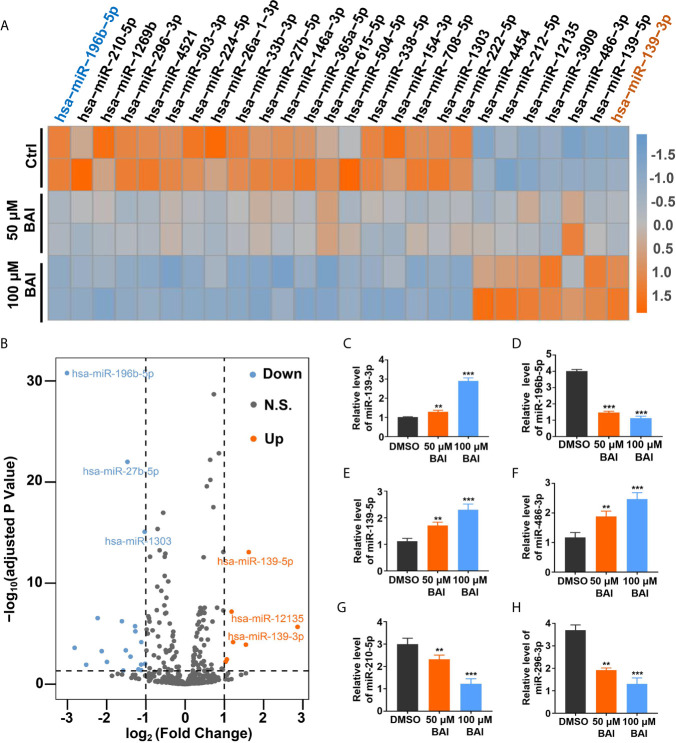
Analysis of miRNAs profiling of Panc-1 cells treated with baicalein. **(A)** Heat map of deferentially expressed miRNAs in Panc-1 cells treated with different dosages of baicalein, log_2_ (Fold Change) >1. **(B)** The volcano plot of deferentially expressed miRNAs. Orange indicates up-regulated expression, grey means no significance and blue denotes down-regulated expression. **(C–H)** Identification of the miRNA level of hsa-miR-139-3p **(C)**, hsa-miR-196b-5p **(D)**, hsa-miR-139-5p **(E)**, hsa-miR-486-3p **(F)**, hsa-miR-210-5p **(G)** and hsa-miR-296-3p **(H)** by qPCR. N.S. means no significance, ** means P < 0.01, *** means P < 0.001.

Previous studies reported that miRNA could bind to mRNAs causing gene silencing and transcriptional inhibition and is responsible for inhibiting tumor cell proliferation, migration and invasion. However, whether baicalein is able to alter several specific miRNAs expression to further influence the progression of pancreatic cancer needs to be further exploited. To clarify the miRNAs pattern in Panc-1 cells after the treatment of baicalein (50 and 100 μM) for 48 h, the miRNA High-Throughput sequencing was performed. The volcano plot showed that 20 miRNAs were up-regulated and 39 miRNAs were down-regulated (**Figure 5B**). The detailed alteration results of miRNAs are shown in **Table S1**. From the heat-map result (**Figure 5A**), we could conclude that the alteration of miRNA expression level was closely related to the concentration of baicalein. Besides, the highest increased miRNA candidate is miR-139-3p (fold change = 7.283) and the highest decreased miRNA candidate is miR-196b-5p (fold change = 0.124) (**Figure 5A**), which were reconfirmed by qPCR (**Figures 5C, D**). Moreover, we also measured the relative miRNA expression of other up-regulated miRNAs (miR-139-5p and miR-486-3p) and down-regulated miRNAs (miR-210-5p and miR-296-3p) (**Figures 5E–H**). In addition, we utilized R-Package clusterProfiler to analyze all of the target genes of miRNAs and draw the diagrams of GO enrichment (**Figures S1A, B**) and KEGG (**Figures S1C, D**). The analysis results indicated that both miR-139-3p and miR-196b-5p participated in multiple essential pathways in pancreatic cancer cells.

### Baicalein Alters the Expression Level of miR-139-3p and miR-196b-5p to Promote Apoptosis

**Figure 6:**
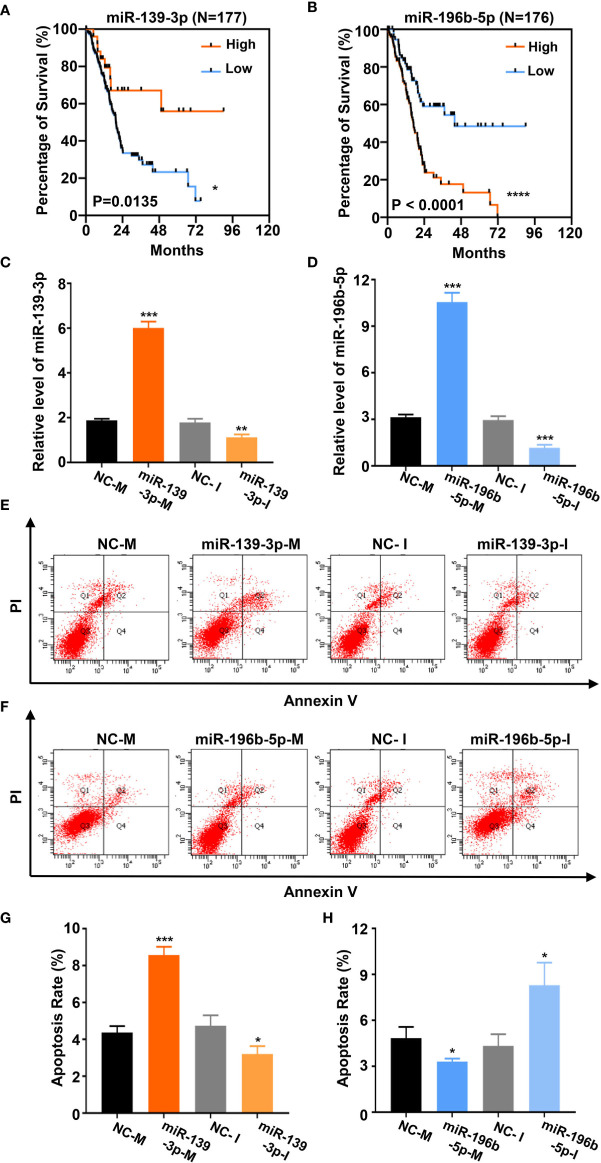
Baicalein alters the expression level of miR-139-3p and miR-196b-5p to promote apoptosis. **(A, B)** Survival curves of 177 **(A)** or 176 **(B)** pancreatic adenocarcinoma samples in TCGA database. (miR-139-3p or miR-196b-5p high-expression group, orange line; miR-139-3p or miR-196b-5p low-expression group, blue line). The number of miR-139-3p or miR-196b-5p high-expression group is 25 or 100. The number of miR-139-3p or miR-196b-5p low-expression group is 152 or 76. **(C, D)** The relative mRNA level of miR-139-3p **(C)** and miR-196b-5p **(D)** transfected with corresponding mimics or inhibitors by qPCR. **(E–H)** The apoptosis rate of miR-139-3p **(E, G)** and miR-196b-5p **(F, H)** transfected with corresponding mimics and inhibitors by flow cytometry. The apoptosis rate equals to the rate of late apoptosis cells (Q2) plus the rate of early apoptosis cells (Q4). * means P < 0.05, ** means P < 0.01, *** means P < 0.001.

**Figure 7:**
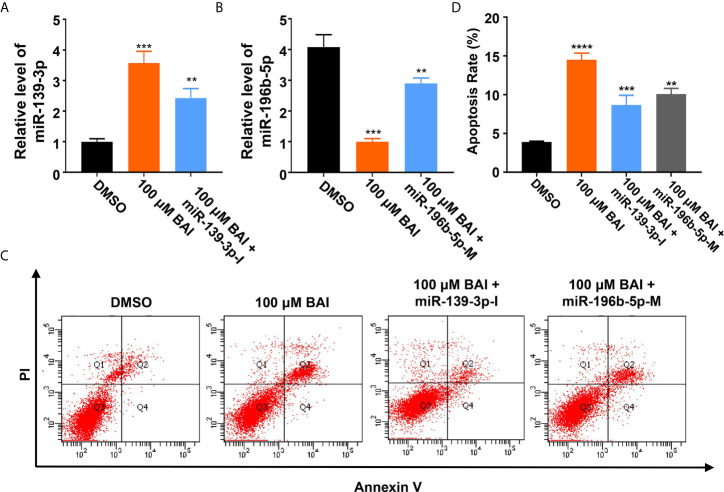
MiR-139-3p-I or miR-196b-5p-M partially rescued the alteration of the expression level of miR-139-3p or miR-196b-5p from baicalein. **(A, B)** The relative mRNA level of miR-139-3p **(A)** and miR-196b-5p **(B)** by qPCR. **(C, D)** The relative apoptosis rate of miR-139-3p and miR-196b-5p in Panc-1 cells treated with DMSO, 100 μM baicalein, 100 μM baicalein and miR-139-3p-I, 100 μM baicalein and miR-196b-5p-M. The apoptosis rate equals to the rate of late apoptosis cells (Q2) plus the rate of early apoptosis cells (Q4). ** means P < 0.01, *** means P < 0.001.

Based on the above analysis data, we determined to clarify whether miR-139-3p and miR-196b-5p play a crucial role in the progression of pancreatic cancer. We firstly analyzed the association of miR-139-3p or miR-196b-5p expression level and the survival percentage of pancreatic cancer patients. Low level of miR-139-3p and high level of miR-196b-5p contribute to the poor survival according to the data from the TCGA (**Figures 6A, B**). To determine the function of miR-139-3p and miR-196b-5p in Panc-1 cells, cells were transfected with miRNA mimics or inhibitors (**Figures 6C, D**). After transfected, the apoptosis rate was measured. The results showed that miR-139-3p mimics (miR-139-3p-M) and miR-196b-5p inhibitor (miR-196b-5p-I) could promote apoptosis, while miR-139-3p inhibitor (miR-139-3p-I) and miR-196b-5p mimics (miR-196b-5p-M) function as inhibiting apoptosis (**Figures 6E–H**). To further explore whether baicalein promotes apoptosis through altering the expression level of miR-139-3p and miR-196b-5p, we performed rescue experiments by transfecting Panc-1 cells with miR-139-3p-I or miR-196b-5p-M after treated with 100 μM baicalein. qPCR results showed that miR-139-3p-I or miR-196b-5p-M partially rescued the alteration of the expression level of miR-139-3p or miR-196b-5p (**Figures 7A, B**). Then we measured the apoptosis rate by flow cytometry (**Figure 7C**), which showed that no matter miR-139-3p-I or miR-196b-5p-M could inhibited the acceleration of apoptosis caused by baicalein (**Figure 7D**).

**Figure 8:**
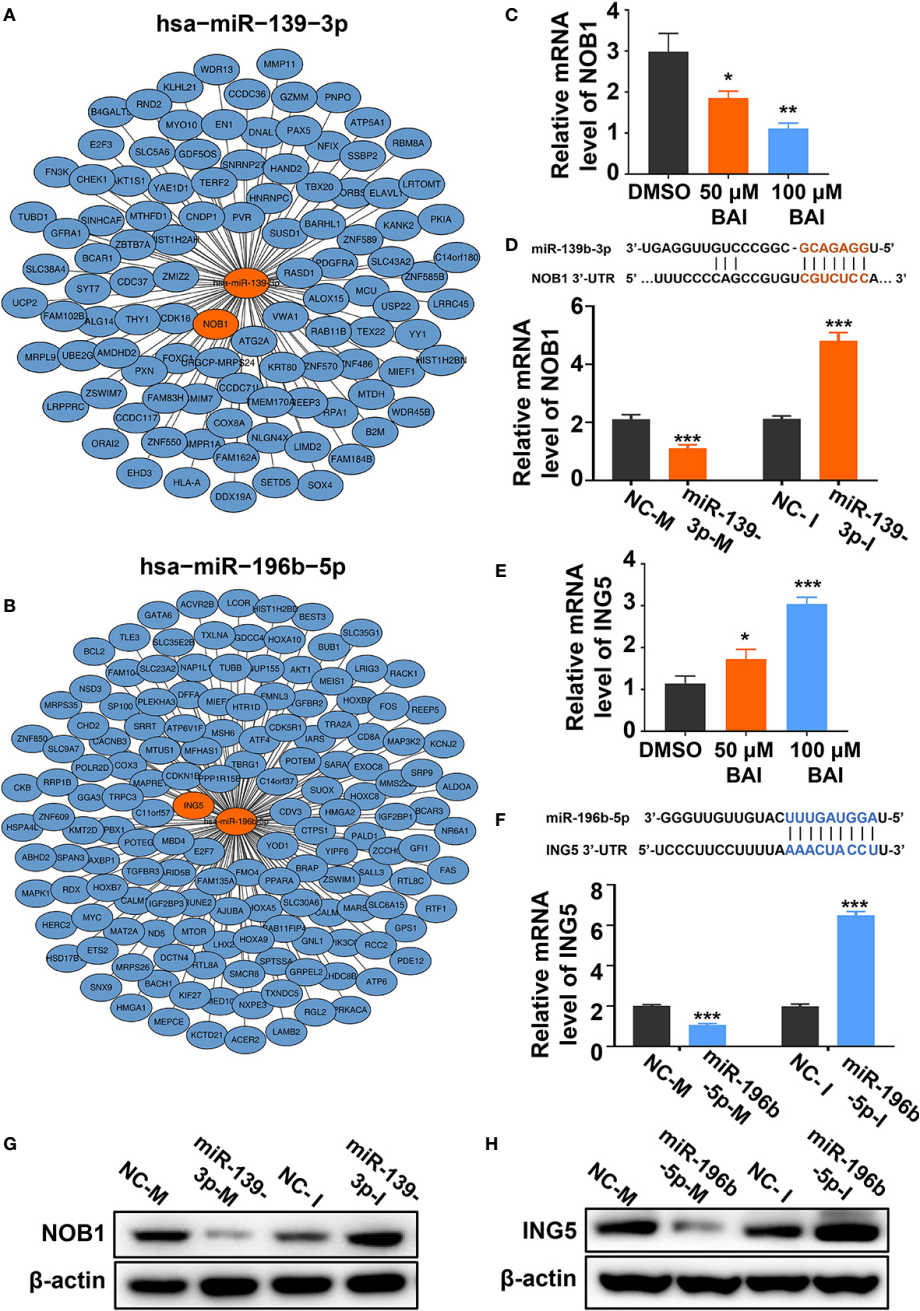
Baicalein decreased the expression level of miR-139-3p downstream NOB1 and increased the expression level of miR-196b-5p downstream ING5. **(A, B)** Interactions between the top-ranked miRNA hsa-miR-139-3p **(A)** or hsa-miR-196b-5p **(B)** with corresponding downstream genes. The target gene of miRNAs was derived from miRTarBase and the network diagram was drawn by Cytoscape. **(C)** The relative mRNA level of NOB1 in Panc-1 cells treated with DMSO, 50 µM and 100 µM baicalein. **(D)** The relative mRNA level of NOB1 transfected with miR-139-3p mimics and inhibitors by qPCR. **(E)** The relative mRNA level of ING5 in Panc-1 cells treated with DMSO, 50 µM and 100 µM baicalein. **(F)** The relative mRNA level of ING5 transfected with miR-196b-5p mimics and inhibitors by qPCR. **(G)** The protein level of NOB1 transfected with miR-139-3p mimics and inhibitors by western blotting. **(H)** The protein level of ING5 transfected with miR-196b-5p mimics and inhibitors by western blotting. * means P < 0.05, ** means P < 0.01, *** means P < 0.001.

We further severally analyzed the relationship between miR-139-3p (**Figure 8A**) or miR-196b-5p (**Figure 8B**) and its down-steamed target genes, which were derived from miRTarBase, and draw the network diagram by Cytoscape. Previous studies showed that miR-139-3p bind to the 3’UTR region of NOB1, which suppresses apoptosis in cancer cells (35). And miR-196b-5p bind to the 3’UTR region of ING5, which induces apoptosis in cancer cells (36). Therefore, we examined the mRNA level of NOB1 and ING5 in Panc-1 cells treated with different dosages of baicalein. Intriguingly, baicalein obviously decreased the expression of NOB1 and increased the expression of ING5 (**Figures 8C, E**). Further, the mRNA and protein level of NOB1 and ING5 were tested respectively after transfected with mimics or inhibitors. The results showed that miR-139-3p-M effectively decreased, whereas miR-139-3p-I increased NOB1 expression (**Figures 8D, G**). Meanwhile, MiR-196b-5p-M decreased, and miR-196b-5p-I increased ING5 expression (**Figures 8F, H**). In conclusion, baicalein plays a key role in promoting apoptosis by up-regulating miR-139-3p or down-regulating miR-196b-5p to alter the expression level of NOB1 and ING5.

## Discussion

In recent years, natural compounds were widely used in many fields including clinical treatment due to their safety and effectiveness. Many preclinical and clinical studies have confirmed that natural compounds have certain therapeutic effects in various diseases, especially cancers (12, 32, 37, 38). Baicalein purified from *Scutellaria baicalensis Georgi* is an active flavonoid ingredient, which was reported participating in inhibiting the progression of various cancers (13–19). However, the detailed mechanism of baicalein acting in pancreatic cancer still remains unclear. In this study, we demonstrated that baicalein plays a critical role in inhibiting pancreatic tumorigenesis *in vitro* and *in vivo.* Our results showed that 100 μM baicalein significantly suppressed the proliferation, motility and migration of pancreatic cancer cells. The Annexin V-FITC/PI assay indicated that baicalein is able to promote the apoptosis, which is similar to the previously reported in breast cancer (17). In order to further explore the mechanism of baicalein promoting apoptosis of pancreatic cancer cells, we analyzed the miRNAs High-Throughput sequencing data. As we expected, baicalein affect the profiling of miRNAs in pancreatic cancer cells. According to the analysis results, miR-139-3p or miR-196b-5p was increased or decreased the most in baicalein treated group. Further verification found that miR-139-3p induced, miR-196b-5p inhibited the apoptosis of Panc-1 cells *via* respectively targeting NOB1 and ING5. Although the concentrations of baicalein exposing to Panc-1 cells were relatively high comparing with some small molecule inhibitors of tumors, the result of *in vivo* experiments showed baicalein nearly has no toxicity to mice. In addition, we chose the appropriate concentrations of baicalein in *in vitro* experiments according to previous studies (11, 13–19, 30, 34, 39, 40).

miRNAs are small endogenous non-coding RNAs, which is able to bind to a variety of specific mRNAs to cause gene silencing. Numerous researchers found that the modulation of miRNAs expression in cancer cells is responsible for activate or inhibit tumorigenesis or metastasis (41). Firstly, miRNAs could directly bind to mRNA to suppress transcription activity of downstream oncogenes or tumor suppressor genes. In the second place, miRNAs also combine with long non-coding RNAs (lncRNAs) through base pairing, which influences transcription activity of downstream oncogenes or tumor suppressor gene (42). Previous studies have showed that baicalein inhibit the progression of lung cancer (13), hepatocellular carcinoma (40) and osteosarcoma (39, 43) by regulating miRNAs. In this study, we proved that baicalein promotes the apoptosis of pancreatic cancer cells by regulating the expression of miR-139-3p and miR-196b-5p. Further, we compared the expression levels of miR-139-3p and miR-196b-5p between normal tissues and pancreatic cancer tissues using TCGA database. Although there are very few miRNA expression data in normal tissues, the difference between the two groups still be seen (**Figures S2A, B**) which indicates that miR-139-3p and miR-196b-5p has a regulatory effect on pancreatic cancer. However, whether baicalein directly regulates the expression of miR-139-3p and miR-196b-5p still needs further exploration. Previously, Yu et al. reported that baicalein inhibits breast cancer growth *via* activating long noncoding RNA (lncRNA) PAX8-AS1-N (34), which indicated that baicalein may indirectly regulate miRNAs by affecting the expression of lncRNAs. In addition, more and more studies have confirmed that A-to-I RNA editing induced by ADARs family enzymes closely related to the tumorigenesis and progression of various types of cancers (44–47). Chen et al.reported that ADARs interact with Dicer to promote the processing of mature miR-27a, which targets a tumor suppressor gene METTL7A (48). Whether baicalein affects the expression of ADARs and regulates the processing of miRNAs is also worthy of further exploration.

In our study, we discovered that baicalein prominently induced cell cycle arrest and promoted apoptosis. Analysis of the High-Throughput sequencing data, we verified that miR-139-3p, the most significant up-regulated miRNA, promotes apoptosis of Panc-1 cells *via* suppressing NOB1 level. Meanwhile, miR-196b-5p, the most significant down-regulated miRNA, restrains apoptosis of Panc-1 cells *via* suppressing ING5 level. Based on existing studies, NOB1 was found to be associated with the 26S proteasome to inhibit apoptosis and ING5 may related to EGFR/PI3K/Akt pathway in colorectal cancer to induce apoptosis. We will further study NOB1 and ING5 participating in which downstream signaling pathways in the future. To sum up, this study not only further confirms the molecular mechanism of baicalein inhibiting pancreatic cancer but also provides a new possibility for the clinical treatment of pancreatic cancer.

## Data Availability Statement

The datasets presented in this study can be found in online repositories. The names of the repository/repositories and accession number(s) can be found below: https://www.ncbi.nlm.nih.gov/sra, PRJNA690773.

## Ethics Statement

The animal study was reviewed and approved by Animal Care and Use Committee, Zhongshan Hospital of Fudan University.

## Author Contributions

DM, SC, HWa and JW conducted the experiments, analyzed data, and wrote the manuscript. HWu, HG, XC and TL analyzed data. YZ and GS provided the concept, designed the study, interpreted the results, and wrote the manuscript. S-HL provided the concept and foundation for revision study, and revised the manuscript. All authors contributed to the article and approved the submitted version.

## Funding

This work was supported by grants from the National Natural Science Foundation of China (No. 81700550 to GS, No. 31502030 to YZ), by the Natural Science Foundation of Shanghai (No. 20ZR1411200 to GS), by the Foundation of Shanghai Municipal Population and Family Planning Commission (No. 20174Y0151 to HW), by the Key Basic Research Program of Science and Technology Commission of Shanghai Municipality (20JC1415300 to HW), by the Foundation of Administration of Traditional Chinese Medicine for Innovative Key Talents of Traditional Chinese Medicine (to SL), by the LOEWE Center Frankfurt Cancer Institute (FCI) funded by the Hessen State Ministry for Higher Education, Research and the Arts (III L 5-519/03/03.001-(0015) to XC).

## Conflict of Interest

The authors declare that the research was conducted in the absence of any commercial or financial relationships that could be construed as a potential conflict of interest.
