# Supplementary material for: Baicalein Induces Apoptosis of Pancreatic Cancer Cells by Regulating the Expression of miR-139-3p and miR-196b-5p
Source: Front Oncol. 2021 Apr 30;11:653061. doi: 10.3389/fonc.2021.653061 (PMC8120266; doi:10.3389/fonc.2021.653061)
Supplement: Supplementary file 1 [file DataSheet_1.pdf]

Supplementary Material

Baicalein induces apoptosis of pancreatic cancer cells by regulating the expression of miR-139-3p and miR-196b-5p

Danhui Ma, Sinuo Chen, Heming Wang, Jiayi Wei, Hao Wu, Hong Gao, Xinlai Cheng, Taotao Liu, Shi-Hua Luo, Yicheng Zhao, Guangqi Song

Supplementary Figures

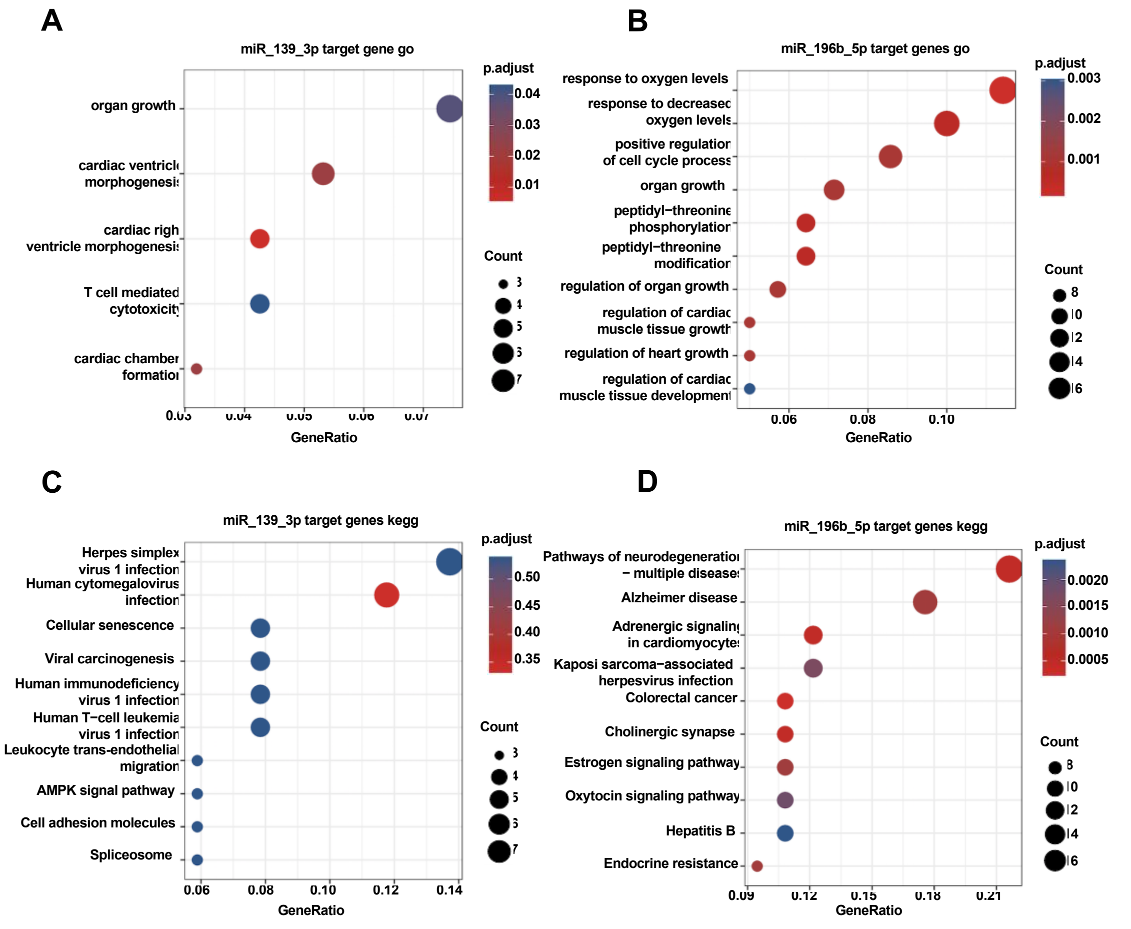

Figure S1. (A-B) GO analysis results of miR-139-3p (A) and miR-196b-5p (B). **(C-D)** KEGG analysis results of miR-139-3p (C) and miR-196b-5p (D).

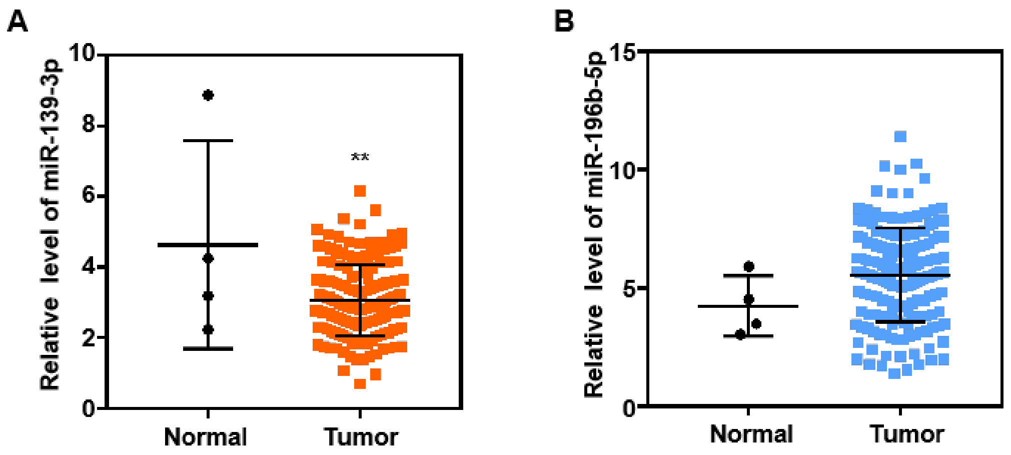

Figure S2. (A) The expression levels of miR-139-3p between normal tissues (n=4) and pancreatic cancer tissues (n=178). **(B)** The expression levels of miR-196b-5p between normal tissues (n=4) and pancreatic cancer tissues (n=178).

Table S1

The alteration of miRNAs in Panc-1 cells treated with 100 μM baicalein. The expression level of 20 miRNAs was up-regulated and 39 miRNAs was down-regulated. Fold Change＞1.5 & baseMean > 10.

miRNA Name	Accession ID	FoldChange	Adjust P-values	Regulation
**hsa-miR-139-3p**	MIMAT0004552	7.282545	2.15736E-06	Up
**hsa-miR-139-5p**	MIMAT0000250	3.082104	8.59986E-14	Up
**hsa-miR-486-3p**	MIMAT0004762	2.924038	0.000127932	Up
**hsa-miR-3909**	MIMAT0018183	2.331688	7.00127E-05	Up
**hsa-miR-12135**	MIMAT0049031	2.275663	6.7525E-08	Up
**hsa-miR-212-5p**	MIMAT0022695	2.090001	0.003654684	Up
**hsa-miR-4454**	MIMAT0018976	2.049308	0.00631957	Up

**hsa-miR-22-5p**	MIMAT0004495	1.964844	8.32658E-14	Up
**hsa-miR-132-5p**	MIMAT0004594	1.962723	5.18452E-08	Up
**hsa-miR-3065-5p**	MIMAT0015066	1.944731	0.037362044	Up
**hsa-miR-1248**	MIMAT0005900	1.910229	0.030070752	Up
**hsa-miR-361-3p**	MIMAT0004682	1.819638	1.44298E-23	Up
**hsa-miR-29b-3p**	MIMAT0000100	1.760884	2.68465E-08	Up
**hsa-miR-22-3p**	MIMAT0000077	1.662964	2.10239E-29	Up
**hsa-miR-182-5p**	MIMAT0000259	1.647463	3.05082E-18	Up
**hsa-miR-362-5p**	MIMAT0000705	1.626103	6.4133E-07	Up
**hsa-miR-877-5p**	MIMAT0004949	1.595598	0.034385343	Up
**hsa-miR-342-3p**	MIMAT0000753	1.589498	0.011979265	Up
**hsa-miR-125b-5p**	MIMAT0000423	1.56387	6.2098E-21	Up
**hsa-miR-30c-5p**	MIMAT0000244	1.55894	6.48165E-23	Up
**hsa-miR-99b-3p**	MIMAT0004678	0.657296	5.74559E-10	Down
**hsa-miR-503-5p**	MIMAT0002874	0.638502	5.75444E-14	Down
**hsa-miR-193b-5p**	MIMAT0004767	0.637751	0.043222108	Down
**hsa-miR-193b-3p**	MIMAT0002819	0.636652	0.000179899	Down
**hsa-miR-210-3p**	MIMAT0000267	0.634739	7.987E-07	Down
**hsa-miR-425-3p**	MIMAT0001343	0.626455	0.002434963	Down
**hsa-miR-1-3p**	MIMAT0000416	0.620036	1.90908E-05	Down

**hsa-miR-27a-5p**	MIMAT0004501	0.61616	4.45364E-16	Down
**hsa-miR-16-2-3p**	MIMAT0004518	0.612021	2.25389E-10	Down
**hsa-miR-7974**	MIMAT0031177	0.607032	4.80325E-08	Down
**hsa-miR-128-1-5p**	MIMAT0026477	0.57635	0.001414609	Down
**hsa-miR-379-3p**	MIMAT0004690	0.563477	4.68781E-06	Down
**hsa-miR-218-5p**	MIMAT0000275	0.538983	2.52908E-13	Down
**hsa-miR-143-3p**	MIMAT0000435	0.535061	0.027188665	Down
**hsa-let-7b-3p**	MIMAT0004482	0.533739	6.4133E-07	Down
**hsa-miR-345-5p**	MIMAT0000772	0.52958	3.06241E-07	Down
**hsa-miR-301a-5p**	MIMAT0022696	0.52279	1.91688E-07	Down
**hsa-miR-410-3p**	MIMAT0002171	0.516407	1.0047E-05	Down
**hsa-miR-23b-5p**	MIMAT0004587	0.507218	3.91835E-06	Down
**hsa-miR-21-3p**	MIMAT0004494	0.50562	0.000487493	Down
**hsa-miR-222-5p**	MIMAT0004569	0.494626	0.00981005	Down
**hsa-miR-1303**	MIMAT0005891	0.48999	8.57718E-16	Down
**hsa-miR-708-5p**	MIMAT0004926	0.461778	6.95494E-05	Down
**hsa-miR-154-3p**	MIMAT0000453	0.46004	0.011845714	Down
**hsa-miR-338-5p**	MIMAT0004701	0.459702	0.043222108	Down
**hsa-miR-504-5p**	MIMAT0002875	0.4389	0.035883542	Down
**hsa-miR-615-5p**	MIMAT0004804	0.416224	5.99476E-06	Down

**hsa-miR-365a-5p**	MIMAT0009199	0.412724	1.83264E-06	Down
**hsa-miR-146a-3p**	MIMAT0004608	0.381238	0.001971732	Down
**hsa-miR-27b-5p**	MIMAT0004588	0.361478	1.00537E-22	Down
**hsa-miR-33b-3p**	MIMAT0004811	0.350795	0.000467711	Down
**hsa-miR-26a-1-3p**	MIMAT0004499	0.335899	0.045190379	Down
**hsa-miR-224-5p**	MIMAT0000281	0.327697	5.96816E-07	Down
**hsa-miR-503-3p**	MIMAT0022925	0.251327	0.006430936	Down
**hsa-miR-4521**	MIMAT0019058	0.229694	0.000570054	Down
**hsa-miR-296-3p**	MIMAT0004679	0.213336	2.89841E-07	Down
**hsa-miR-1269b**	MIMAT0019059	0.174222	0.012201299	Down
**hsa-miR-210-5p**	MIMAT0026475	0.142066	0.000260448	Down
**hsa-miR-196b-5p**	MIMAT0001080	0.124141	1.71912E-31	Down

**TABLE S2 | ** The sequence of qPCR primers used in this paper.

Primer name	Sequence (5'-3')
**GAPDH-F**	AGCCACATCGCTCAGACAC
**GAPDH-R**	GCCCAATACGACCAAATCC
**U6-F**	CTCGCTTCGGCAGCACA
**U6-R**	AACGCTTCACGAATTTGCGT
**Caspase-3-F**	TGCCTGTAACTTGAGAGTAGATGG
**Caspase-3-R**	CTTCACTTTCTTACTTGGCGATGG
**p21-F**	TGTCTTGTACCCTTGTGCCTC
**p21-R**	TGGTAGAAATCTGTCATGCTGGT
**CCND1-F**	GAGGAGCTGCTGCAAATGGA
**CCND1-R**	GGAGGGCGGATTGGAAATGA
**miR-139-3p-F**	TCGGCAGGTGGAGACGCGGCCCTGT
**miR-139-3p-R**	GCAGGGTCCGAGGTATTC
**miR-139-5p-F**	TCTACAGTGCACGTGTCTCCAGT
**miR-139-5p-R**	TGGAGACACGTGCACTGTAGATT
**miR-486-3p-F**	GCGGGGCAGCTCAGTA
**miR-486-3p-R**	CGGGGCAGCTCAGTACAGGAT
**miR-196-5p-F**	GCGCGTAGGTAGTTTCCTGTT
**miR-196-5p-R**	AGTGCAGGGTCCGAGGTATT
**miR-296-3p-F**	GCCGAGGAGGGTTGGGTGGA
**miR-296-3p-R**	CTCAACTGGTGTCGTGGA
**miR-210-5p-F**	CCTGCAATATTTGCATGTCG
**miR-210-5p-R**	GTCCCTATTGGCGTTACTATGG
**NOB1-F**	ATCTGCCCTACAAGCCTAAAC
**NOB1-R**	TCCTCCTCCTCCTCCTCAC
**ING5-F**	GCACAAAGGAGGGTCTGA

**ING5-R**	TGGGTTTCGTGGTAAGGT
